# Complete response to disitamab vedotin in HER2-low metastatic endometrial carcinoma: a case report and review of the literature

**DOI:** 10.3389/fonc.2024.1367140

**Published:** 2024-09-16

**Authors:** Hu Feng, Shasha Bi, Shanshan Sun, Hongbo Yang, Haoxing Zhou, Jingjing Mao, Na Li, Fujun Yang

**Affiliations:** ^1^ Department of Oncology, Weihai Municipal Hospital, Cheeloo College of Medicine, Shandong University, Weihai, Shandong, China; ^2^ Department of Pathology, Weihai Municipal Hospital, Cheeloo College of Medicine, Shandong University, Weihai, Shandong, China; ^3^ Shenzhen Engineering Center for Translational Medicine of Precision Cancer Immunodiagnosis and Therapy, YuceBio Technology Co., Ltd, Shenzhen, China

**Keywords:** endometrial cancer, HER2-low, ADCS, RC48, recurrent metastasis, complete response

## Abstract

Endometrial cancer (EC) is one of the most common gynecologic malignancies with increasing morbidity. The prognosis for patients diagnosed with early-stage EC remains favorable; however, for patients with recurrent or metastatic EC, the prognosis is poor and treatment options, until recently, are limited. Antibody drug conjugates (ADCs) represent innovative strategies in cancer treatment; however, there are less investigations regarding their efficacy in EC. This report describes an EC case with low human epidermal growth factor receptor 2 (HER2) immunohistochemistry (IHC) expression score (IHC 2+) that experienced recurrent metastasis in the abdominal and peritoneal following post-surgical chemotherapy and radiotherapy. Subsequently, the commencement of HER2-targeted ADC, disitamab vedotin (RC48; 2.5 mg/kg), administered intravenously every two weeks, was initiated. The tumor lesions shrunk markedly after three cycles of treatment and disappeared by the completion of ten cycles of therapy. The patient is still in remission at present. The current findings imply the potential efficacy of HER2-targeted ADCs for patients with HER2-low metastatic EC.

## Introduction

Endometrial carcinoma (EC) is one of the most common gynecological cancer worldwide, with rising incidence and mortality rates (1). Clinically, EC is classified into two types based on Bokhman typing system, namely type I and type II (2). Type I EC is typically hormone sensitive and has an good prognosis, with the endometrioid carcinoma being the prevailing histological subtypes, whereas type II EC is non-hormone-dependent and has a tendency to recur, even in early stage, mainly including plasmacytoma, clear cell carcinoma, and carcinosarcoma (3). The standard initial treatment for EC involves a hysterectomy, bilateral salpingo-oophorectomy, and assessment of the retroperitoneal lymph nodes (4). Following the identification of four distinct molecular subtypes of EC by The Cancer Genome Atlas, their impact on prognosis has prompted the recommendation for treatment considerations based on subtype (5, 6). For EC cases that have previously been treated, the US Food and Drug Administration (FDA) has approved pembrolizumab and dostarlimab as immune checkpoint inhibitors (ICIs). Additionally, for cases that are mismatch repair-proficient and microsatellite-stable, pembrolizumab in combination with lenvatinib is an approved treatment option for those who have progressed after treatment with at least one previous systemic therapy (7).

Antibody drug conjugates (ADCs) are designed to selectively achieve their desired cell targets while sparing healthy tissues, thereby reducing systemic toxicities (8). While the approval of ADCs specifically for EC is yet to be achieved, a multitude of them are actively undergoing thorough evaluation (9). HER2, a receptor tyrosine-protein kinase, emerges as a promising target for therapeutic intervention in the realm of EC. The loss of HER2 expression is common in metastatic EC lesions, underscoring the significance of determining the potential benefit of anti-HER2 therapies in EC patients (10). Disitamab vedotin (RC48) is a newly developed HER2-targeted ADC coupling of hertuzumab with monomethyl auristatin E via a cleavable linker (11). RC48 has garnered approval for its effective application in the treatment of cancer patients with HER2-overexpressing (IHC2+/3+) locally advanced or metastatic gastric carcinoma/gastric and gastroesophageal junction carcinoma (GC/GEJC) as well as urothelial carcinoma (UC) who have been treated with systemic chemotherapy agents at least twice (12). In the RC48-C011 study (13), for HER2-negative (IHC 0/1+) advanced UC patients treated with ≥1 prior systemic therapy, RC48 also showed promising efficacy, with an objective response rate of 26.3%. RC48 has also exhibited promising efficacy in a phase I study for both HER2-positive and HER2-low breast cancer (14, 15). As there is no standardized scoring system for HER2 expression in EC, the use of ADCs targeted HER2 could be widely beneficial. However, there are no clinical trials and reports addressing the efficacy and safety of RC48 in EC.

This report details the case of a 69-year-old female patient diagnosed with endometrial clear-cell carcinoma (Type II EC), characterized by negative programmed death ligand 1 (PD-L1) expression, low MSI score, low tumor mutational burden (TMB), and low HER2 expression. After experiencing a metastasis in the abdominal and peritoneal region within 10 months following surgery, adjuvant chemotherapy, and radiotherapy, the patient underwent RC48 treatment. After completing 10 cycles of RC48 therapy, the recurrent tumor lesions in the abdominal and peritoneal regions completely disappeared, and the patient currently remains in a state of complete remission (CR).

## Results

### Case presentation

**Figure 1:**
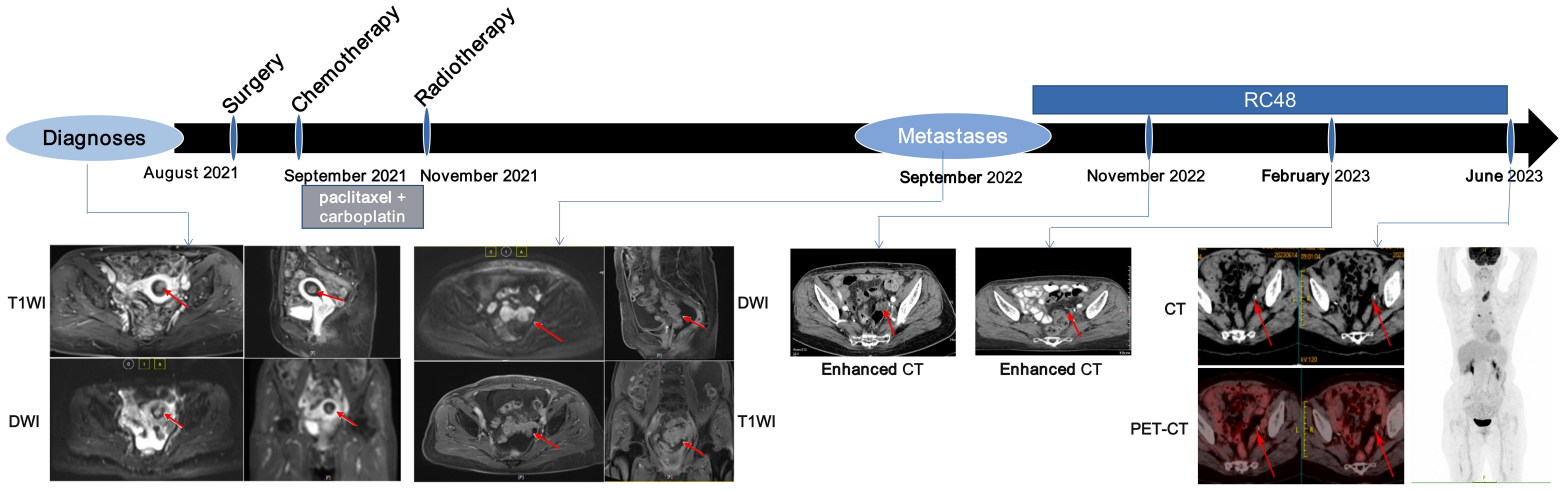
Course of the disease with treatment history and images. Red arrows show the location of tumor lesions.

In early August 2021, a 69-year-old woman who had been in menopause for 18 years was admitted to the hospital with irregular vaginal bleeding for 10 days. The patient underwent transcervical resection of endometrium and dilatation and curettage. Pathology in combination with abdominal and pelvic magnetic resonance imaging (MRI) examination confirmed that the tumor was an endometrial clear cell carcinoma (Type II EC) with a size of 1.5×0.9×0.3 cm^3^ (**Figure 1**; **Supplementary Table 1**).

Histopathological examination was performed and the IHC exhibited estrogen receptor (ER)-, progesterone receptor (PR)-, MSH2 (+), MSH6 (+), MLH1 (+), PMS2 (+), HER2 (2+), and Ki-67 proliferation marker (Ki-67) (30-40%) (**Supplementary Figure 1**). Based on the 2018 endometrial clinical trial fluorescence *in situ* hybridization (FISH) evaluation criteria, tumors were assessed as *HER2* FISH negative (**Supplementary Figure 2**). Following the guidelines of the American Joint Committee on Cancer, 8th edition (16), the patient’s tumor node metastasis (TNM) classification was determined as pT3aN0M0, indicating stage IIIA.

In late August 2021, the patient underwent laparoscopic total extrafascial hysterectomy, bilateral adnexectomy, pelvic lymph node dissection, and paraaortic lymph node resection. Following the surgical procedure, the patient received adjuvant chemotherapy, which involved the administration of four cycles of albumin paclitaxel plus carboplatin starting in September 2021 (**Supplementary Table 1**). Throughout this treatment, the patient experienced severe bone marrow suppression (grade IV) and gastrointestinal reactions (grade III). In November 2021, postoperative adjuvant radiotherapy (External radiation therapy: Three dimensional adaptive intensity-modulated radiation therapy (IMRT) with PTV = 2Gy × 23F = 46Gy; Internal irradiation: Iridium-192 high-dose rate after close range treatment: 6Gy × 2F = 12Gy) was conducted for the patient (**Supplementary Table 1**).

In September 2022, abdominopelvic MRI revealed the presence of abdominal and peritoneal metastases (**Figure 1**). Due to the patient’s prior history of severe adverse reactions following postoperative adjuvant chemotherapy, as well as the short interval between the completion of adjuvant radiotherapy and the recurrence, the patient does not meet the criteria for chemotherapy-palliative radiotherapy as outlined in the Chinese Guidelines for the Diagnosis and Treatment of Endometrial Cancer (v2022).

IHC staining demonstrated low PD-L1 expression (tumor proportion score < 1%) for the primary tumor (**Supplementary Table 2**). NGS data indicated low TMB (2.01 muts/Mb) and MSI score (4.70) for the primary tumor (**Supplementary Table 2**). Moreover, blood samples obtained at metastases also indicated a low bTMB (1.0 muts/Mb) and bMSI score (5.72) (**Supplementary Table 2**). Collectively, these results indicated that the patient was unlikely to benefit from immunotherapy. Additionally, due to the unavailability of tumor tissues from metastatic lesions, cells derived from abdominal fluid were collected and made into wax blocks for HER2 IHC, which detected a low level of HER2 expression (IHC 1+) (**Supplementary Figure 3**). Since anti-HER2 ADCs have been reported to be effective in *HER2*-low expression cancers (13, 15, 17, 18), the patient was recommended the cross-indication anti-HER2 ADC, RC48 (initially at a dosage of 60 mg, followed by 120 mg every two weeks). After 3 cycles of treatment, the computed tomography (CT) scan showed significantly reduced in the pelvic metastatic lesion by 65% (**Figure 1**). There has been a notable decrease in carbohydrate antigen 125 (CA-125) level (**Supplementary Figure 4**). After 10 cycles of treatment, CT examination unveiled the vanishing of multiple metastatic lesions (**Figure 1**), while the CA-125 level returned to its normal range (**Supplementary Figure 4**). The patient’s response to treatment was assessed using the RECIST 1.1 guidelines, which determined CR. In June 2023, positron emission tomography-CT examination revealed a sustained stability without any signs of progression (**Figure 1**). The CA-125 level remained within the normal range (**Supplementary Figure 4**). The evaluation of efficacy continued to demonstrate a CR. The patient experienced grade II bone marrow suppression (manifested as leukopenia) during treatment. There were no serious adverse reactions during the application of RC48 treatment. Until August 2024, the patient had maintained an excellent response with no progression signs.

## Discussion

In this case, the patient had type II EC with an initial tumor stage of T3N0M0. HER2 tumor expression at diagnosis and at time of recurrence was IHC (2+)/FISH-negative and IHC (1+), respectively. The patient consented to try domestically developed ADC drug-RC48 and exhibited a favorable response. To our knowledge, this is the first report of a metastatic EC with low HER2 expression showing a marked response to ADCs, thus underscoring the potential viability of targeted therapy utilizing ADCs as a favorable treatment option for metastatic EC with low HER2 expression.

Although the FDA has yet to approve any ADCs specifically for EC, there are several promising candidates currently under investigation. Among them, a contender focuses on targeting the overexpression of FRα receptors, which are present in approximately 64% of endometrial tumors. An ongoing phase II trial (ClinicalTrials.gov identifier: NCT03835819) aims to evaluate the efficacy of mirvetuximab soravtansine in microsatellite-stable EC. Additionally, another agent called STRO-002, is currently undergoing assessment in a phase I trial (ClinicalTrials.gov identifier: NCT03748186) for EC. HER2, a receptor tyrosine-protein kinase, has shown promising potential as a therapeutic target in EC treatment. Trastuzumab-deruxtecan (T-DXd), an ADC designed to target HER2, has already received approval for HER2-positive and HER2-low breast cancer (17, 18) and is being investigated for its efficacy in HER2-positive EC in the clinical trial DESTINY-PanTumor02 (ClinicalTrials.gov identifier: NCT04482309). Furthermore, T-DXd demonstrated anti-tumor efficacy and durable responses in heavily pretreated patients across various tumor types with activating *HER2* mutations, with no new safety signals observed in the clinical trial DESTINY-PanTumor01 (ClinicalTrials.gov identifier: NCT04639219). Hence, prespecified HER2 mutations might be targeted through HER2-directed ADCs. Additionally, a study (ClinicalTrials.gov identifier: NCT04585958) is currently underway to assess the combined use of T-DXd and olaparib (PARPi) specifically in HER2-positive serous EC. Another promising candidate, DB-1303, an ADC composed of an anti-HER2 monoclonal antibody, a cleavable peptide-linker, and a topoisomerase I inhibitor, has obtained fast track designation from the FDA. Preclinical models have demonstrated DB-1303’s favorable antitumor activity and safety profiles in both HER2-positive and HER2-low tumors. It is Currently being evaluated in an ongoing phase I/IIa trial (ClinicalTrials.gov identifier: NCT05150691) for patients with advanced/unresectable, recurrent, or metastatic EC. Disitamab vedotin (RC48) is another innovative anti-HER2 ADC that combines hertuzumab (a novel anti-HER2 monoclonal antibody) with monomethyl auristatin E (MMAE) through a cleavable linker. RC48 has gained approval from the National Medical Products Administration (NMPA) for the treatment of locally advanced or metastatic gastric cancer/gastroesophageal junction carcinoma with HER2 overexpression (11). However, there is currently no available data regarding the effectiveness of RC48 in patients with EC.

Further investigation is needed to fully understand the mechanism by which anti-HER2 ADCs exhibit their efficiency in cancers with low HER2 expression. Preclinical studies have shown that activating mutations in *HER2* enhance receptor internalization and intracellular uptake of the complex formed by the HER2 receptor ADC complex (19). This finding may explain the observed effectiveness in *HER2*-mutant cancers where HER2 expression is undetectable, as indicated by an IHC score of 0.

A recent study has made a significant discovery, highlighting the remarkable potential of combining RC48 with PD-1/PD-L1 inhibitors to greatly enhance tumor suppression and strengthen the body’s immune response against tumor (20). This synergistic approach has been observed to promote extensive infiltration of T cells and activation of immune markers in a human syngeneic breast cancer model that expresses HER2. Furthermore, this combination therapy has demonstrated the capacity to induce the generation of immune memory in animal subjects, leading to complete tumor eradication and long-lasting protection against tumor recurrence (21). Previous research has suggested that ADC drugs possess inherent antitumor immune activity by exerting a potent cytotoxic impact on tumor cells or adjacent tissues following the intracellular release of highly effective cytotoxic payload (22, 23). An example of such a payload is MMAE (24), a powerful inhibitor of tubulin polymerization and a key component of RC48 (25). Therefore, the simultaneous administration of RC48 along with anti-PD-1/PD-L1 antibodies holds great potential as an emerging therapeutic strategy for patients with EC in forthcoming clinical trials.

This case presented certain limitations. Firstly, the difficulty in obtaining tissue samples during recurrence posed a challenge. Consequently, well-established immunotherapy biomarkers like PD-L1 expression and mismatch repair deficiency were not assessed prior to initiating treatment with RC48. Secondly, there remains uncertainty regarding the change in HER2 status between the primary and recurrent disease, as the cells extracted from abdominal fluid were processed into wax blocks for HER2 IHC. Lastly, larger cohorts are required to gain a more comprehensive elucidation of the underlying mechanism. Therefore, we will enroll such EC patients to further investigate the efficacy of RC48 and explore the underlying mechanism.

## Conclusion

In summary, we have reported a remarkable response in an EC patient with HER2 low expression (IHC 1+) when treated with RC48 following metastasis. While our report is based on a single case, it highlights the effectiveness of anti-HER2 drugs in metastatic EC. RC48 exhibits promising potential in HER2-low metastatic EC and warrants further investigation in patients with low HER2 expression, as well as evaluation of its efficacy in front-line therapy.

## Methods

### Targeted next-generation sequencing

The formalin-fixed paraffin-embedded (FFPE) tumor tissue slides, along with corresponding blood samples, were dispatched to YuceBio, a biotechnique laboratory in Shenzhen, China, authenticated by the College of American Pathologists (CAP). The samples underwent NGS utilizing the YuceOne™ Pro extensive targeted panel, which encompasses 1,021 genes. The extraction of genomic DNAs was performed using the GeneRead DNA FFPE Kit (Qiagen) for tumor tissue samples, and the DNA blood mini kit (Qiagen) for the blood samples. To ensure data quality, sequencing reads with a > 10% N rate and/or > 10% bases with a quality score < 20 were filtered using OAPnuke (V1.5.6) (26).

### Evaluation of genomic biomarkers

TMB was defined as the number of all nonsynonymous mutations and indels per megabase of the genome analyzed. TMB > 10 muts/Mb was categorized as TMB-High (TMB-H). The MSI status was determined using MSIsensor (V0.2) (27). Subsequently, an in-house tool was employed to reevaluate and rectify the MSI value. The MSI sensor score represented the percentage of unstable sites. MSI scores ≥ 20 were identified as MSI-High.

### Immunohistochemistry

The IHC staining assay was conducted on FFPE tissue sections, following the guidelines provided by manufacturers. PD-L1 expression was evaluated using the Dako PD-L1 IHC 22C3 pharmDx assay. The measurement of PD-L1 expression was based on the tumor proportion score (TPS), which calculated the percentage of tumor cells displaying complete or partial membrane staining in the central or marginal tumor region. PD-L1 expression levels were categorized as “low” if TPS < 1% and as “high” if TPS was ≥ 50%, adhering to standard recommendation found in previous publications (28). Additionally, IHC was performed for HER2 (anti-HER2/neu (4B5) rabbit monoclonal antibody; VENTANA), ER (anti-ER SP1 rabbit monoclonal antibody; VENTANA), PR (anti-PR 1E2 rabbit monoclonal antibody; VENTANA), MSH2 (ZA-0622; anti-MSH2 RED2 rabbit monoclonal antibody; ZSGB-Bio), MSH6 (ZA-0541; anti-MSH6 EP49 rabbit monoclonal antibody; ZSGB-Bio), MLH1 (ZM-0154; anti-MLH1 ES05 mouse monoclonal antibody; ZSGB-Bio), PMS2 (ZA-0542; anti-PMS2 EP51 rabbit monoclonal antibody; ZSGB-Bio), and Ki67 (ZM-0166; anti-Ki67 UMAB107 mouse monoclonal antibody; ZSGB-Bio). IHC scoring for HER2 followed the 2018 ASCO/CAP guidelines (29) as follows: 0: No staining or ≤ 10% of infiltrating tumor cells show incomplete and weak cell membrane staining; 1+: More than 10% of infiltrating tumor cells show incomplete and weak cell membrane staining; 2+: More than 10% of infiltrating tumor cells exhibit weak to moderate intensity intact cell membrane staining; ≤ 10% of infiltrating tumor cells exhibit strong and complete cell membrane staining; 3+: More than 10% of infiltrating tumor cells exhibit strong and complete cell membrane staining. IHC 3+ is judged as HER2 positive, while IHC 0 and 1+ are judged as HER2 negative. IHC 2+ patients are further subjected to *in situ* hybridization for HER2 gene amplification status detection.

### Fluorescence *in situ* hybridization

The PathVysion assay (Abbott Molecular, Inc, Abbott Park, Illinois) was utilized for HER2 FISH analysis, adhering to the manufacturer’s instructions. This analysis was performed in conjunction with HER2-immunostained slides, focusing on the region displaying the most pronounced HER2 protein expression. The pathology FISH procedure reports provided the HER2/CEP17 signal ratio, HER2 copy number per nucleus, CEP17 copy number per nucleus, and the count of tumor cells. The interpretation of the FISH results followed the 2018 endometrial clinical trial criteria, represented a modified version of the ASCO/CAP 2007 breast criteria (29).

## Data Availability

The datasets presented in this study can be found in online repositories. The names of the repository/repositories and accession number(s) can be found below: https://www.ncbi.nlm.nih.gov/, PRJNA1156245.

## Supplementary material

Supplementary Figure 1 IHC staining of the primary tumor. (down panel magnification, ×100). Scale bar: 400 μM. IHC, immunohistochemistry; HER2, human epidermal growth factor receptor 2; ER, estrogen receptor; PR, progesterone receptor.

Supplementary Figure 2 *HER2* gene amplification by fluorescence *in situ* hybridization (FISH) with *HER2*/CEP17 ratio of 1.11, *HER2* copy number of 3.10, and CEP17 copy number of 2.80. The left and right panels are two duplicates. *HER2* signal: red, CEP17 signal: green. Magnification: ×1000. Scale bar: 20 μM.

Supplementary Figure 3 Immunohistochemical stain showed a low HER2 protein expression (IHC 1+) after recurrence. Left panel: magnification, ×40; Scale bar: 1000 μM. Right panel: magnification, ×100; Scale bar: 400 μM.

Supplementary Figure 4 Clinical outcomes of CA125 level. The CA125 level decreases after surgical resection of the primary lesion, but dramatically increases during recurrence and returns to normal levels after RC48 treatment. CA125, carbohydrate antigen 125.
