# Supplementary figures and images for: Complete response to disitamab vedotin in HER2-low metastatic endometrial carcinoma: a case report and review of the literature
Source: Front Oncol. 2024 Sep 16;14:1367140. doi: 10.3389/fonc.2024.1367140 (PMC11439626; doi:10.3389/fonc.2024.1367140)

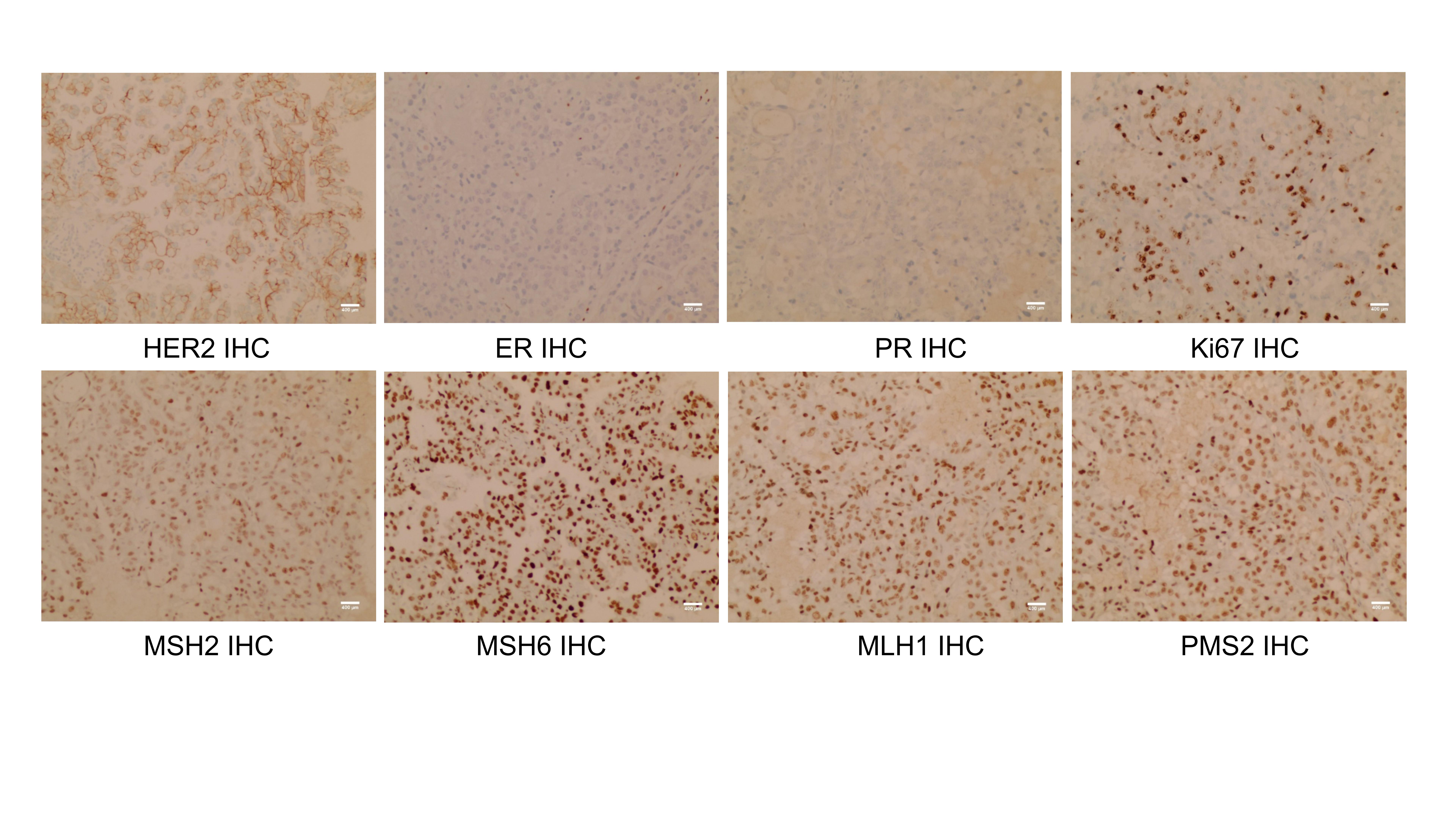

Supplement: Supplementary Figure 1 — IHC staining of the primary tumor. (down panel magnification, ×100). Scale bar: 400 μM. IHC, immunohistochemistry; HER2, human epidermal growth factor receptor 2; ER, estrogen receptor; PR, progesterone receptor. [file Image1.jpeg]

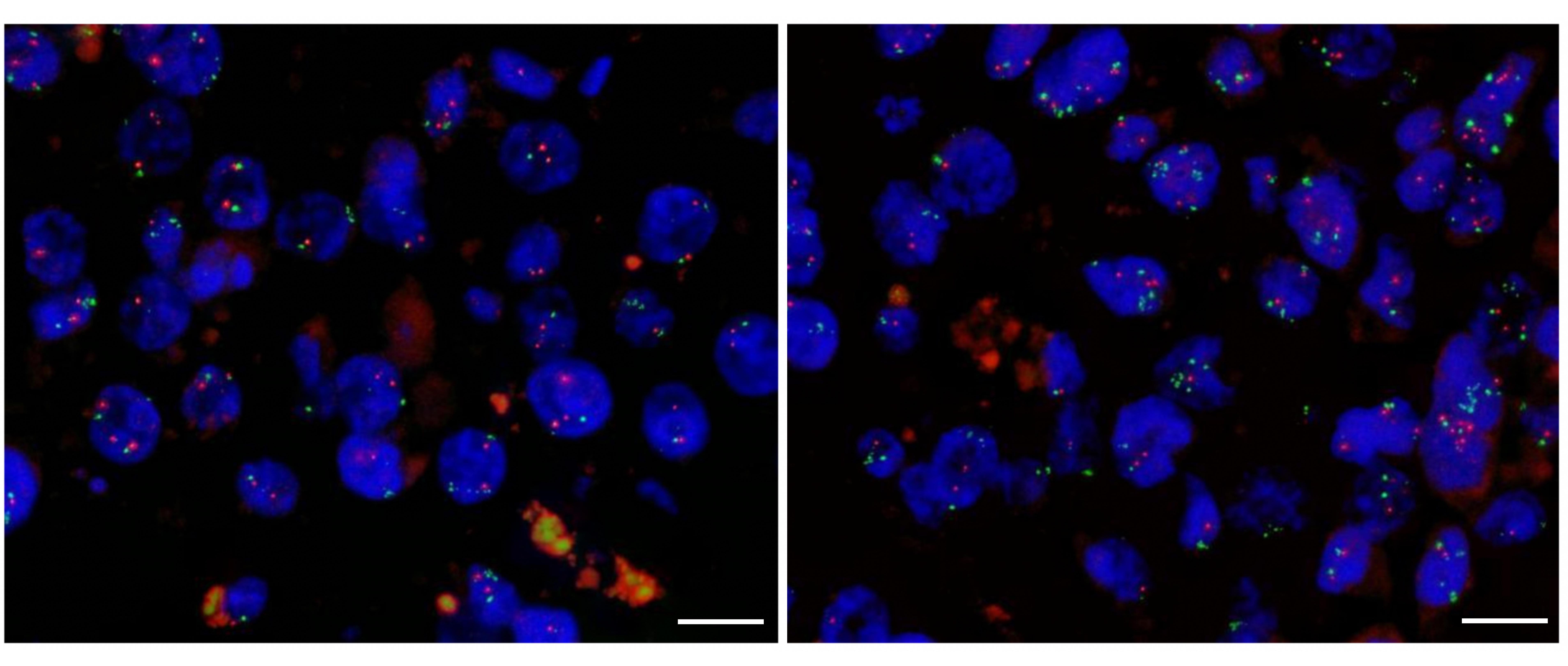

Supplement: Supplementary Figure 2 — HER2 gene amplification by fluorescence in situ hybridization (FISH) with HER2/CEP17 ratio of 1.11, HER2 copy number of 3.10, and CEP17 copy number of 2.80. The left and right panels are two duplicates. HER2 signal: red, CEP17 signal: green. Magnification: ×1000. Scale bar: 20 μM. [file Image2.jpeg]

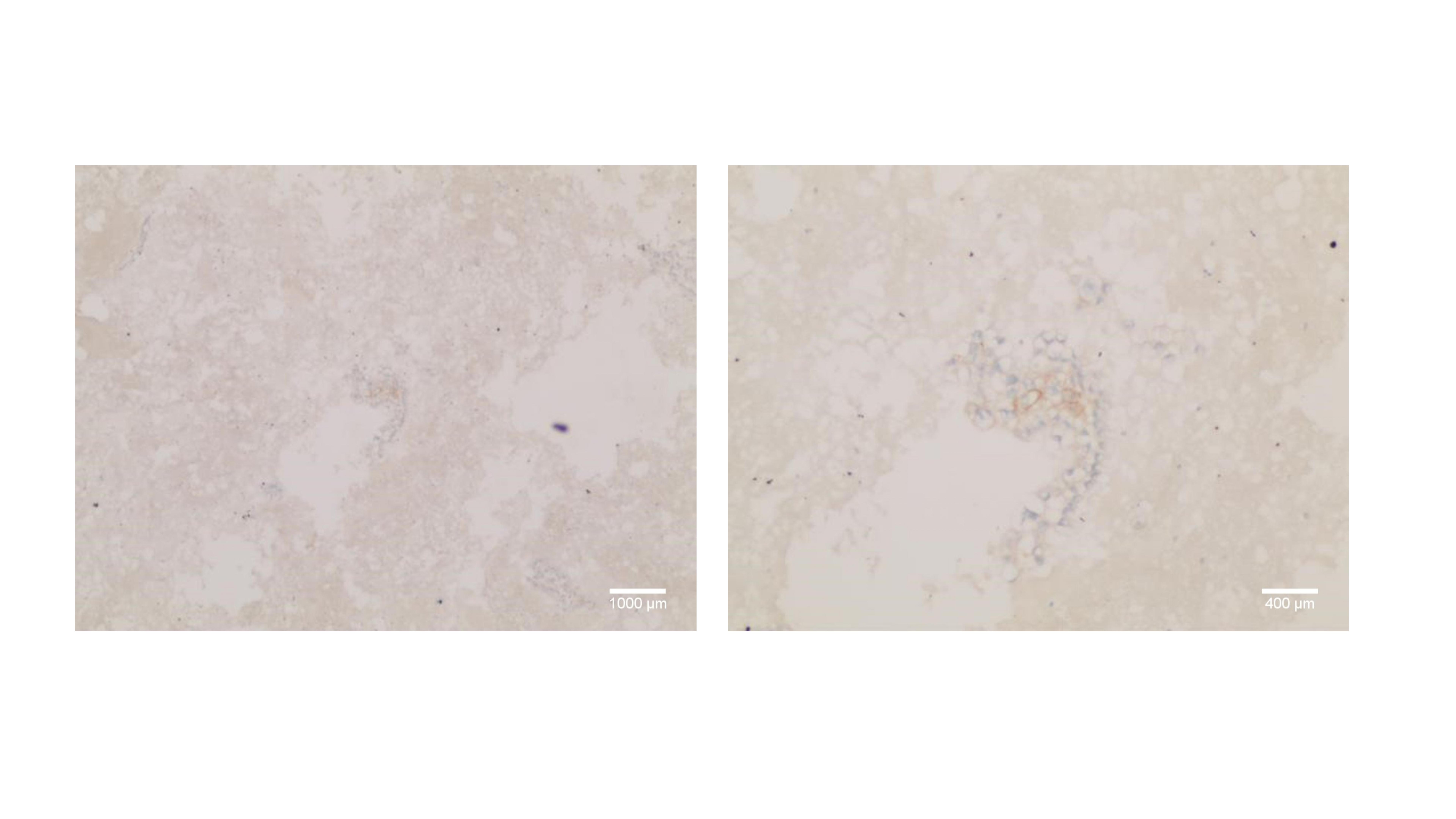

Supplement: Supplementary Figure 3 — Immunohistochemical stain showed a low HER2 protein expression (IHC 1+) after recurrence. Left panel: magnification, ×40; Scale bar: 1000 μM. Right panel: magnification, ×100; Scale bar: 400 μM. [file Image3.jpeg]

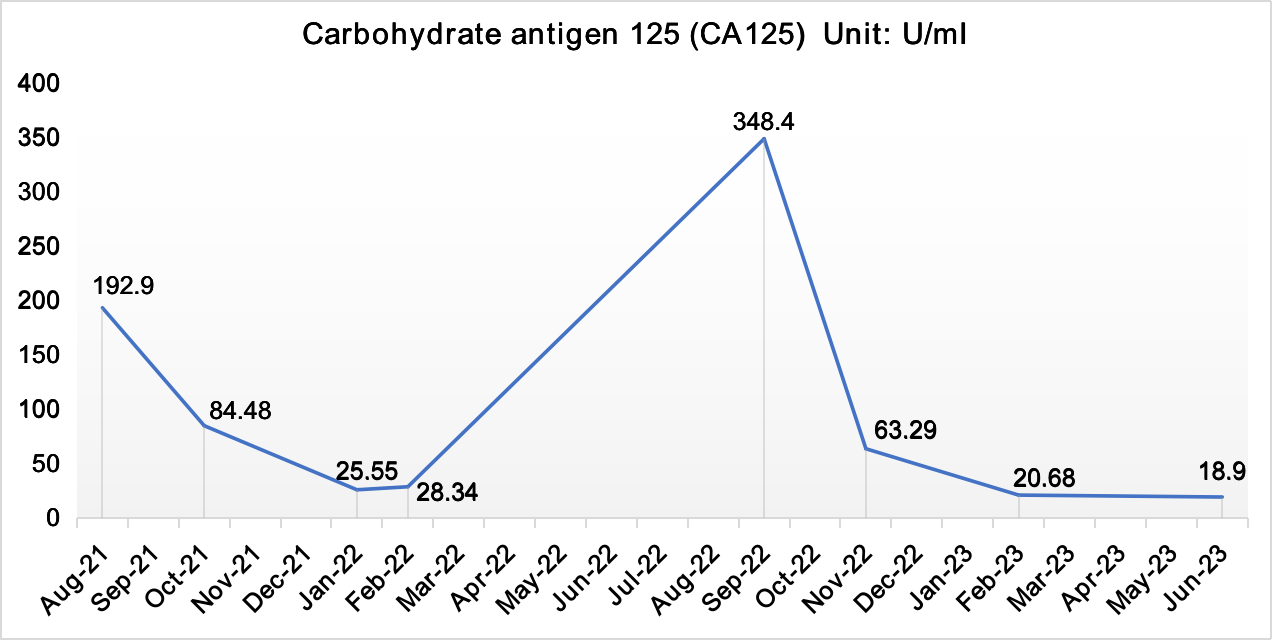

Supplement: Supplementary Figure 4 — Clinical outcomes of CA125 level. The CA125 level decreases after surgical resection of the primary lesion, but dramatically increases during recurrence and returns to normal levels after RC48 treatment. CA125, carbohydrate antigen 125. [file Image4.jpeg]
