# Supplementary material for: Complete response to disitamab vedotin in HER2-low metastatic endometrial carcinoma: a case report and review of the literature
Source: Front Oncol. 2024 Sep 16;14:1367140. doi: 10.3389/fonc.2024.1367140 (PMC11439626; doi:10.3389/fonc.2024.1367140)
Supplement: Supplementary file 5 [file Table1.docx]

**Supplementary Table 1. Detailed information on the disease diagnosis and treatment.**

|  | August 26, 2021 | September 29, 2021 | November 8, 2021 | September 1, 2022 | November 9, 2022 | February 15, 2023 | June 14, 2023 |
| --- | --- | --- | --- | --- | --- | --- | --- |
| **Treatment** | Operation:  laparoscopic extrafascial total hysterectomy + bilateral adnexectomy + pelvic lymph node dissection + abdominal paraaortic lymph node dissection. | started postoperative adjuvant chemotherapy (albumin paclitaxel 300 mg, day 1, four cycles, Q2W plus carboplatin, 400 mg day 2, four cycles, Q2W). | Radiotherapy:  1) In vitro radiotherapy dose: DT46Gy/23fx;  2) Brachytherapy: DT12Gy/2fx. | 1) Confirm recurrence and metastasis  2) September 26, 2022  RC-48 drug treatment 60 mg for the first time, 120 mg Q2W for each subsequent time, | After 1.5 months of RC48 treatment | After 5 months of RC48 treatment | After 9 months of RC48 treatment |
| **Information** | The histopathological examination revealed that the endometrial clear cell carcinoma was confined to the mucosal layer and did not extend to the lower uterine segment. There was no evident presence of intravascular cancer thrombus or nerve invasion. Additionally, cancerous tissue was observed on the surface of the right ovary, while no cancerous tissue was identified on the cervix, left ovary, and bilateral fallopian tubes.  **Immunohistochemical result:**  ER(-), PR(-), Vimentin(-), P53(wild-type), Ki67 (+, 30-40%), MSH2(+), MSH6 (+), (MLH1+), PMS2 (+), HER2 (2+), HNF1B (+), Napsin (partial +), p504s (+) | Throughout this treatment, the patient experienced severe bone marrow suppression (grade IV) and gastrointestinal reactions (grade III). |  | Multiple nodular lesions, characterized by low T1WI, high T2WI, and high DWI signals, were detected in various locations, including the vesico-rectal recesses, paracolic sulci, omentum, mesentery, etc. The larger lesions were about 6.5 cm × 3.1 cm and displayed infiltration into the pelvic wall. Additionally, dilation was observed in the left ureter and renal pelvis. Furthermore, multiple small lymph node shadows as well as many fluid-like signal shadows were observed in the pelvic cavity. | Upon conducting a Pelvic CT examination, a low-density focus measuring approximately 2.3 cm in length was discovered around the iliac vessel in the left pelvic wall. This focus exhibited slight enhancement at its border. Moreover, multiple small lymph nodes were observed within the pelvic and inguinal regions. Notably, no obvious enhancement was observed in the pelvic cavity during the enhanced scan. Additionally, a small presence of liquid density shadow was noticed on the pelvic floor. | The CT examination revealed low density surrounding the iliac vessel in the left pelvic wall, exhibiting decreased clarity compared to previous observations. Within the pelvic cavity and groin area, multiple small lymph nodes can be seen. Furthermore, there were no abnormal enhancement in the pelvic cavity during the enhanced scan. | The PET-CT scan showed no significant abnormal enlargement in the abdominal cavity or retroperitoneum, and there are no increased FDG uptake in the lymph nodes. Following the surgery for endometrial cancer, no abnormal thickening or increased FDG uptake was detected in the uterus and bilateral adnexa. A small, slightly dense nodule measuring approximately 1.3 cm in diameter was observed in the left perineum area, with decreased FDG uptake. In the right lower abdominal mesenteric region, there were small flaky density shadows and small lymph nodes in the right external iliac vascular shape area. No abnormal increase in FDG uptake was noted. Additionally, there were no abnormal lymph nodes observed in the left iliac vascular circulation area or bilateral inguinal regions. |
