# Supplementary material for: Complete response to disitamab vedotin in HER2-low metastatic endometrial carcinoma: a case report and review of the literature
Source: Front Oncol. 2024 Sep 16;14:1367140. doi: 10.3389/fonc.2024.1367140 (PMC11439626; doi:10.3389/fonc.2024.1367140)
Supplement: Supplementary file 6 [file Table2.docx]

**Supplementary Table 2. Overview of TMB, MSI, and PD-L1 levels.**

| **Sample** | **TMB** | **TMB_Type** | **MSI** | **MSI_Type** | **PD-L1 TPS** | **PD-L1_Type** | **PD-L1 CPS** | **PD-L1_Type** |
| --- | --- | --- | --- | --- | --- | --- | --- | --- |
| **Primary tumor tissue** | 2.01 | TMB-L | 4.70 | MSI-L | < 1 | PD-L1 low | < 1 | PD-L1 low |
| **Blood collected at recurrence** | 1.0 | TMB-L | 5.72 | MSI-L | / | / | / | / |

TMB, tumor mutation burden; MSI, microsatellite instability; PD-L1, programmed cell death 1 ligand 1; TPS, tumor proportion score; CPS, combined positive score.
